# A South African Public-Private Partnership HIV Treatment Model: Viability and Success Factors

**DOI:** 10.1371/journal.pone.0110635

**Published:** 2014-10-20

**Authors:** Jude Igumbor, Sophie Pascoe, Shuabe Rajap, Wendy Townsend, John Sargent, Ernest Darkoh

**Affiliations:** 1 Research and Development Department, BroadReach Healthcare, Cape Town, South Africa; 2 Operations Department, BroadReach Healthcare, Cape Town, South Africa; 3 BroadReach Healthcare, Cape Town, South Africa; Vanderbilt University, United States of America

## Abstract

**Introduction:**

The increasing number of people requiring HIV treatment in South Africa calls for efficient use of its human resources for health in order to ensure optimum treatment coverage and outcomes. This paper describes an innovative public-private partnership model which uses private sector doctors to treat public sector patients and ascertains the model’s ability to maintain treatment outcomes over time.

**Methods:**

The study used a retrospective design based on the electronic records of patients who were down-referred from government hospitals to selected private general medical practitioners (GPs) between November 2005 and October 2012. In total, 2535 unique patient records from 40 GPs were reviewed. The survival functions for mortality and attrition were calculated. Cumulative incidence of mortality for different time cohorts (defined by year of treatment initiation) was also established.

**Results:**

The median number of patients per GP was 143 (IQR: 66–246). At the time of down-referral to private GPs, 13.8% of the patients had CD4 count <200 cell/mm^3^, this proportion reduced to 6.6% at 12 months and 4.1% at 48 months. Similarly, 88.4% of the patients had suppressed viral load (defined as HIV-1 RNA <400 copies/ml) at 48 months. The patients’ probability of survival at 12 and 48 months was 99.0% (95% CI: 98.4%–99.3%) and 89.0% (95% CI: 87.1%–90.0%) respectively. Patient retention at 48 months remained high at 94.3% (95% CI: 93.0%–95.7%).

**Conclusions:**

The study findings demonstrate the ability of the GPs to effectively maintain patient treatment outcomes and potentially contribute to HIV treatment scale-up with the relevant support mechanism. The model demonstrates how an assisted private sector based programme can be effectively and efficiently used to either target specific health concerns, key populations or serve as a stop-gap measure to meet urgent health needs.

## Introduction

With advances in antiretroviral treatment and improved survival of people living with HIV, the South African health system has to contend with an unprecedented demand for HIV/AIDS-related health care resources. Preliminary reports of the South African household HIV prevalence survey already suggest an almost 2% increase in prevalence between 2010 and 2012 [1]. This scenario excludes the continued HIV transmission and increase in the number of people who will require treatment following the new WHO treatment initiation recommendations [2], [3]. These factors exacerbate the need for more efficient use of South Africa’s total human resources for health to meet the growing demand for treatment.

In spite of the above, there has been an astronomical increase in the number of people living with HIV who are on treatment in South Africa [4]. Estimates show that the number of people on antiretroviral therapy in South Africa increased from less than 600,000 in 2008 to over 2,100,000 in 2013 [4], [5]. However, a significant proportion of people requiring treatment still do not have access to it, with the treatment gap estimated to be as high as 45% in certain populations [3]–[5]. In addition, the current ART coverage success is fraught with challenges of poor patient retention, suboptimal adherence and treatment failure, partly due to insufficient human resources and high patient load for the limited capacities in health facilities [6]–[9]. Halting and reversing this challenge will require continued innovations to further enhance access to treatment and reduce patient load per service provider. In this regard, it became necessary to explore other options, one of which is to harness skills in the private sector to help address this national challenge with far reaching consequences.

Against this background, this paper describes the outcomes of an innovative public-private-partnership (PPP) model in an attempt to ascertain its ability to maintain patient treatment outcomes and the model’s success factors that can be harnessed for future programmes. The model – referred to as the private general medical practitioner (GP) model – assumes the treatment of stable patients who were originally initiated on treatment at public health facilities.

## Methodology

The GP model was developed at the request of the North West Provincial Department of Health. The GP model is one of the three models used by BroadReach Healthcare (BRHC) – a private healthcare solutions company – to provide HIV treatment through selected private GPs. The GP model was designed to be sustainable with minimal external financial support. The costs supported by BroadReach Healthcare included the start-up costs of setting up a staffed office within selected public sector wellness clinics, patient data management, adherence support and a negotiated consultation fee for the private GPs. The Department of Health provided ART medication from their district pharmacy, and laboratory services were rendered by National Health Laboratory Services which is a government parastatal. Eligible patients were down-referred to a select group of GPs in the communities where the patients live. The GPs were selected based on their prior training and experience in HIV/AIDS treatment. Patients were considered eligible for down referral if they had been stable on ART for at least 3–6 months with a suppressed viral load, did not have private medical insurance, did not have concurrent illnesses (excluding TB) and voluntarily consented to participate in the programme. The patients were then cared for by the private GPs as an alternative to public sector health service outlets. The patients were initially referred back to the public sector for the management of acute co-morbidities and complications. This was changed after 2012 to allow the GPs to manage co-morbidities. The adherence and treatment support services provided by BroadReach Healthcare were in the form of patient education sessions and adherence/wellness training, which patients attended upon their down-referral and on an annual basis. Other adherence support services provided by BroadReach Healthcare included the use of clinic-based adherence supporters; home visits by adherence supporters and telephonic follow up.

**Figure 1:**
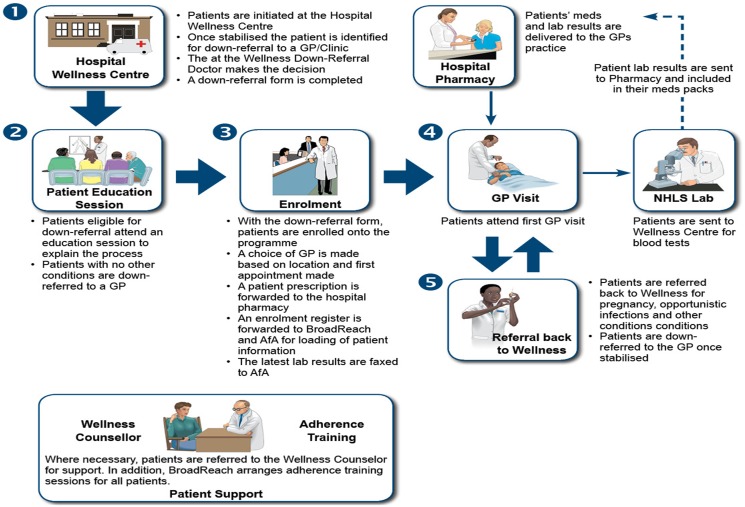
Model implementation process and components.

A key component of the model was its patient information management system, referred to as the Disease Management System (DMS). BroadReach Healthcare’s DMS is managed by Aid for AIDS (AfA) which is a private data company that is funded through donor funds. The DMS promotes efficient management of complex patient information; it is available both online and offline (for remote use); and provides real-time patient information such as treatment regimen and pick-up history, laboratory information, doctors’ consultation notes and adherence support data. The DMS is also automated to send important phone text messages, to remind patients, doctors, pharmacists and laboratory personnel of treatment activities. The reminders included scheduled treatment pick-up and laboratory test dates, details of defaulting patients, undesirable laboratory results and uncollected treatments. Some of these innovations were not part of the routine public sector treatment programme. Figure 1 illustrates the model’s implementation processes and scope of services offered. Further information about the model is available from the authors.

This study was based on a retrospective design and used the electronic records of patients enrolled in the GP model. Patients included in this analysis are cared for by 40 GPs. The database used for the study was developed for patient management but contained the minimum scope of variables needed for this study’s objectives. The data elements taken from the database for this study were age, sex, treatment initiation dates, treatment regimen, results of routine clinical tests and respective test dates of the patients. Patients younger than 18 years were rarely recruited into the GP model and were thus excluded from the analysis. Outcome variables considered were viral load, CD4 cell counts and patient retention. The treatment outcomes were categorized, for example, viral load status was defined as either suppressed or unsuppressed using 400 copies/ml as the status threshold for suppression. Methods of CD4 count and viral load testing have been described [10]. Loss-to-follow-up in the study was defined as patients on treatment who stopped picking up their treatment and have had no contact with the treatment programme for three months or more after their last scheduled visit date was missed. The deceased and lost-to-follow-ups were further anonymously verified to be actually lost-to-follow-up and not deceased using the national death records. Time in the study for the deceased and those lost-to-follow-up was defined as the time of initiation into the GP model to the last contact date. Data was statistically analysed using STATA 11. The median CD4 count and proportion of patients with suppressed viral load (defined as HIV-1 RNA less than 400 copies/ml) were calculated at baseline and at 6, 12, 24 and 48 months. Cumulative incidence of mortality for different time cohorts (defined by year of treatment initiation) was also established. Prior data of modest quality could not be obtained hence the baseline data referred to in this paper is the data at the point of down referral into the GP model. The median time to death and attrition was established, followed by the calculation of the survival functions for mortality and attrition respectively.

The study is based on data that is collected and stored on DMS (which is managed by a different private company on behalf of BroadReach Healthcare). The database used for this study had no individual patient identifier and the analysis was completely anonymous. Patients in the GP model also signed a consent for their information to be entered into the central database and used for routine decisions on patient and programme management and were made aware that any further analyses will exclude patient identifiers.

## Results

**Figure 2:**
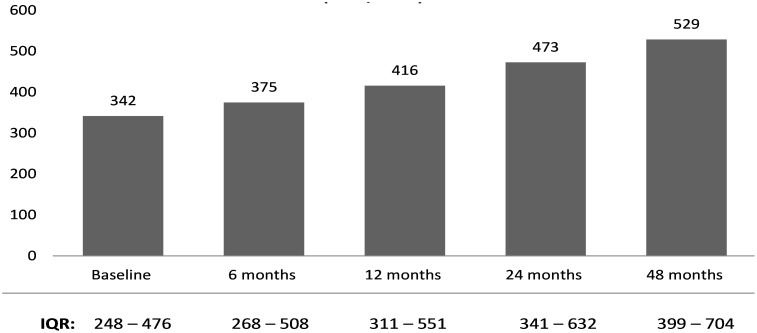
Median CD4 cell counts (cells/mm^3^) from baseline at down-referral to 48 months in the GP model.

The database used for this analysis included 2535 longitudinal patient records from their respective time of down-referral to November 2012. The first group of patients were down-referred into the partnership model in November 2005. The patients’ duration in the care of the GPs ranged from 2–84 months with a 28 months median. The median number of patients each GP ever managed in the model was 143 (IQR: 66–246). The median age of the patients was 40 years old (IQR 34–46). About two-thirds (68.4%) of the patients were female. The median CD4 cell count at baseline was 342 cells/mm^3^ (IQR 248–476). Figure 2 shows that this indicator increased progressively for those who remained in the programme. The median viral load over this period however remained the same (25, IQR 25–40).

**Figure 3:**
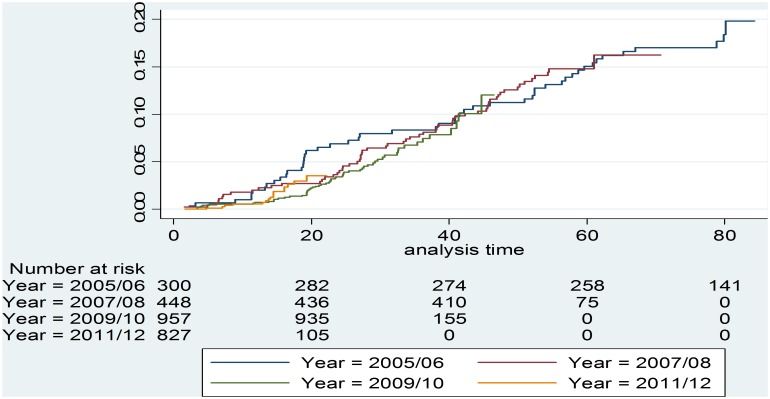
Cumulative incidence of mortality by year of treatment initiation.

The GP model showed similar treatment outcome when the probability of survival of cohorts initiated to treatment at different times for a minimum of 60 months (five years) using logrank test (p>0.05) was compared. Figure 3 shows the cumulative incidence of mortality across the treatment initiation cohorts by year of treatment initiation.

**Figure 4:**
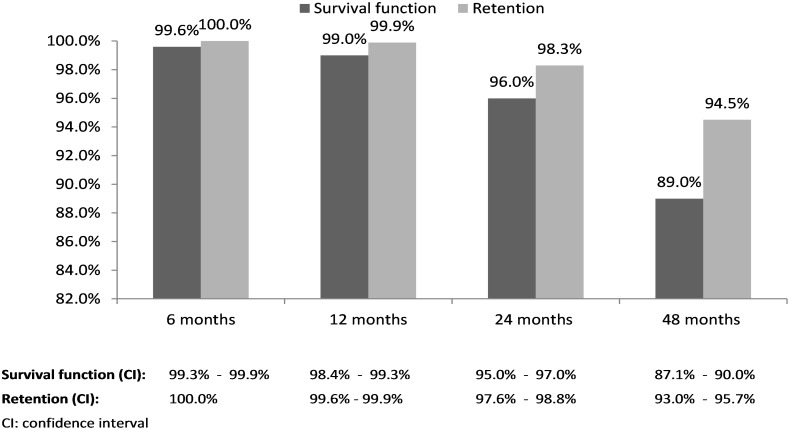
Survival and retention rates at different points.

The survival functions at six (6) and 48 months are 99.6% (95% CI: 99.3%–99.9%) and 89.0% (95% CI: 87.1%–90.7%) respectively. The retention function at six (6) and 48 months are 100% and 94.5% (95% CI: 93.0%–95.7%) respectively. Survival and retention rates at 6, 12, 24 and 48 months are also shown in Figure 4. The median time to death for patients who died in the model was 21 months (IQR: 12.3–38.1) and the median time to attrition for patients who were lost to follow up was 29 months (IQR: 19.7–45.7).

## Discussion

The study findings demonstrate the ability of the private sector to successfully absorb and retain public sector patients over time. The GP model therefore presents an opportunity to significantly reduce the growing burden of HIV/AIDS care on the public sector. Leveraging more GPs for public sector patients or marginally decreasing doctor-patient ratio could further improve treatment coverage, decongest the public sector and obtain good patient retention and treatment outcomes [8], [11].

Unlike most HIV/AIDS programmes, this model only caters for patients who have been stabilized through public sector programmes. This makes it difficult to compare its outcomes with the outcomes of other programmes that enrol all patients irrespective of their viral load. Whilst a direct comparison may be spurious, patient progression noted after down-referral into the GP model is similar to the progression among patients initiated to treatment at CD4 cell counts of between 350 cells/mm^3^ and 500 cells/mm^3^ [12]. Other studies have found linkages between treatment outcome and year of treatment initiation with improvements in successive years [13]. This trend has been associated with the advances in ART and improved access to services. This study noted significant differences when probability of survival was disaggregated into groups based on their year of enrolment into the GP model. This pattern may have resulted from improvements in psychosocial adjustment to living with HIV and adherence to treatment over time among people who have been in programme for a long time [14]. This proposition may also be a factor of progressively improved immunologic stability and durability of virologic suppression with time [15]–[17].

The successes reported in this study may also be attributed to the combination of established strengths of both public and private sector HIV/AIDS programmes. The GP model on the one hand harnesses public sector experience and regulations, and on the other leverages the private sector human resources and infrastructure to ensure patient retention and decongestion of public sector facilities. With this, the GP model proposes a complementary model that harnesses the arguments of both the proponents of universal state-based health care and the advocates of using the private sector to provide health services in areas where the public sector does not have capacity [18].

The private sector in general is often criticized for its high cost and inefficiencies in terms of unnecessary diagnostic tests and treatment options [18]. To address these, patient treatment and testing in the GP model was provided by government pharmacies and laboratories using standard government guidelines. An earlier cost-effectiveness study on this model found it to be relatively cost-effective when compared to down-referring patients to public sector clinics [19]. Concerns about the quality of care in the private sector have also been raised by previous studies [20], [21]. Other studies have found diagnostic inaccuracies and sub-therapeutic clinical management to be more profound in the private sector in comparison to the public sector [22]–[26]. In this regard, the GP model is guided and monitored using government regulations and other support mechanisms including quarterly meetings with public hospital clinicians to discuss cases. These measures and the electronic support systems help to better assure adherence to the prescribed minimum diagnostic and therapeutic guidelines [25]. A combination of adherence support measures were used in the model. The use of such multiple adherence support strategies is also known to improve and better guarantee adherence to treatment and patient retention both of which are critical for optimum treatment outcome [6], [27]. Treatment discontinuation due to stock-out has been shown to result in treatment interruption and increased risk of death [28]–[30]. The GP model’s data management system promotes efficient stock management and treatment availability and this is pivotal in dealing with the common drug shortages in the public sector [31].

The limitation of this study was its exclusion of patients transferred-out of the GP programme for a variety of reasons. Following-up patients who have been transferred-out of a programme remains a major problem for many treatment programmes [32]. Consequently, the large scale of the South African epidemic and treatment programme plus the vital importance of uninterrupted treatment and patient retention should necessitate solutions that track individual patients across different points of service uptake and treatment programmes.

In conclusion, the GP model demonstrates the ability of an assisted private sector treatment programme to retain patients and maintain immunologic and virologic outcomes. This model offers lessons to guide strategic and operational decisions, specifically on how to use private sector resources to target defined health concerns or key populations or serve as a stop-gap measure to meet urgent health needs.
